# Targeted Editing of the *pp38* Gene in Marek’s Disease Virus-Transformed Cell Lines Using CRISPR/Cas9 System

**DOI:** 10.3390/v11050391

**Published:** 2019-04-26

**Authors:** Yaoyao Zhang, Jun Luo, Na Tang, Man Teng, Vishwanatha R.A.P. Reddy, Katy Moffat, Zhiqiang Shen, Venugopal Nair, Yongxiu Yao

**Affiliations:** 1 The Pirbright Institute & UK-China Centre of Excellence for Research on Avian Diseases, Pirbright, Surrey GU24 0NF, UK; zhangyaoyao3848@126.com (Y.Z.); tangna0543@163.com (N.T.); Vishi.AvalakuppaPapiReddy@pirbright.ac.uk (V.R.A.P.R.); Kathryn.Moffat@pirbright.ac.uk (K.M.); 2 College of Animal Science and Technology, Guangxi University, Nanning 530004, China; 3 Key Laboratory of Animal Immunology of the Ministry of Agriculture, Henan Provincial Key Laboratory of Animal Immunology, Henan Academy of Agricultural Sciences, Zhengzhou 450002, China; luojun593@aliyun.com (J.L.); tm135@aliyun.com (M.T.); 4 College of Animal Science and Technology, Henan University of Science and Technology, Luoyang 471003, China; 5 Binzhou Animal Science and Veterinary Medicine Academy & UK-China Centre of Excellence for Research on Avian Diseases, Binzhou 256600, China; bzshenzq@163.com; 6 The Jenner Institute, University of Oxford, Old Road Campus Research Building, Roosevelt Drive, Oxford OX3 7DQ, UK; 7 Department of Zoology, University of Oxford, 11a Mansfield Road, Oxford OX1 3SZ, UK

**Keywords:** CRISPR/Cas9, MDV-transformed cell line, pp38, GFP, proliferation

## Abstract

Marek’s disease virus (MDV), a lymphotropic α-herpesvirus associated with T-cell lymphomas in chickens, is an excellent model for herpesvirus biology and virus-induced oncogenesis. Marek’s disease (MD) is also one of the cancers against which a vaccine was first used. In the lymphomas and lymphoblastoid cell lines (LCLs) derived from them, MDV establishes latent infection with limited gene expression. Although LCLs are valuable for interrogating viral and host gene functions, molecular determinants associated with the maintenance of MDV latency and lytic switch remain largely unknown, mainly due to the lack of tools for in situ manipulation of the genomes in these cell lines. Here we describe the first application of CRISPR/Cas9 editing approach for precise editing of the viral gene phosphoprotein 38 (*pp38*), a biomarker for latent/lytic switch in MDV-transformed LCLs MDCC-MSB-1 (Marek’s disease cell line MSB-1) and MDCC-HP8. Contradictory to the previous reports suggesting that pp38 is involved in the maintenance of transformation of LCL MSB-1 cells, we show that *pp38*-deleted cells proliferated at a significant higher rate, suggesting that *pp38* is dispensable for the transformed state of these cell lines. Application of CRISPR/Cas9-based gene editing of MDV-transformed cell lines in situ opens up further opportunities towards a better understanding of MDV pathogenesis and virus-host interactions.

## 1. Introduction

Marek’s disease (MD) is a lymphoproliferative disease of chickens with a complex pathogenesis, characterized by neoplastic transformation of T cells that infiltrate lymphoid tissues, visceral organs, and even peripheral nerves. Marek’s disease virus (MDV) is highly contagious and chicks get infected via the respiratory route by inhalation of poultry dust from the contaminated poultry houses. Once infected, MDV undergoes a cytolytic infection of B and T cells in lymphoid organs followed by establishment of latency in CD4+ T cells and integration of its genome into the telomeres of host chromosomes [1,2,3]. The integration process is very efficient as up to 15 chromosomes in tumor cells has been observed harboring the MDV genome detected by either fluorescent in situ hybridization (FISH) or pulsed-field gel electrophoresis (PFGE) [2,4,5,6,7]. Due to factors or events that are still largely unknown, some of the latently infected CD4+ T cells in susceptible and unvaccinated birds subsequently get transformed into neoplastic cells, resulting in the development of lymphomas, leading to high levels of mortality [8,9]. The precise trigger(s) for neoplastic transformation are not known, although major viral genes associated with transformation have been identified.

Following the development of both overlapping cosmid clones and bacterial artificial chromosome (BAC) technologies, the function of several MDV genes has been investigated in vitro and in infection models in the natural chicken hosts [10,11,12,13,14,15,16,17,18,19,20,21,22,23]. Among the viral determinants of oncogenicity, the basic leucine zipper protein Meq (MDV *Eco*RI Q) is considered to be the most important and the most extensively studied. Deleting the *Meq* gene or abolishing some of the important interactions such as CtBP (C-terminal binding protein) affected the oncogenicity of the virus [10,18,24]. Similarly, MDV-encoded microRNA MDV-miR-M4 and viral telomerase RNA (vTR) have also been shown to play a significant role on MDV-induced oncogenesis [25,26,27,28]. Although the application of BAC and overlapping cosmid technologies have enabled significant progress in our understanding of the disease and the virus, a number of major features of this complex disease are yet to be revealed such as the latency, transformation, and host-virus interaction. Thus far, most of the data on MDV gene expression during the neoplastic stages of the disease have come from lymphoblastoid cell lines (LCL) derived from MD lymphomas. As clonal populations of transformed tumor cells with latent MDV genome and limited gene expression [29,30,31], LCLs provide an extremely valuable source to study the latency, reactivation, and transformation in situ. However, the manipulation of the viral and host genes in these cell lines hitherto has been challenging primarily because of the lack of availability of efficient tools. Robust gene editing technologies based on the CRISPR/Cas9 system have revolutionized bioscience research providing the capability for deleting, mutating, or inserting genes for interrogating gene functions in many different contexts including virus-transformed cancer cell lines. For example, CRISPR/Cas9 has been used successfully for genome engineering of Epstein-Barr virus (EBV) transformed LCLs for functional knock-out of target gene protein expression [32] and microRNA [33], genome-wide loss-of-function screens [34], detection of DNA regulatory elements [35], and clear latent virus infection [33].

We have recently demonstrated that avian herpesvirus genomes can be efficiently edited using the CRISPR/Cas9 system for gene function studies as well as recombinant vaccine development [36,37]. While these studies have been carried out in cell culture systems in vitro that supports lytic virus replication, we wanted to examine whether the latent MDV genome in transformed LCLs can be manipulated using CRISPR/Cas9 editing system for gaining further insights into host-virus interactions during latency and lytic switch. MDV-encoded phosphoprotein pp38, strongly associated with lytic replication of the virus in B cells, is thought to play an important role in maintaining the transformed status of lymphocytes in vivo by preventing apoptosis, although its role in reactivation has been shown to be debatable [38]. Previously, we have reported deletion of *pp38* from the vaccine strain CVI988 using CRISPR/Cas9 editing [39] in infected CEF (primary chick embryo fibroblasts). In this report, we have used a new approach with the same gRNA sequences to delete *pp38* and insert green fluorescent protein (GFP) into pp38 in MDV-transformed LCLs MSB-1 and HP8 to examine its functional roles. Continued proliferation of the pp38 knock-out cell lines confirmed that the *pp38* gene is not essential for maintenance of the transformed state of these cell lines. This report on the first successful application of the CRISPR/Cas9-based gene editing technology on MDV-transformed LCLs in situ will open the door for more targeted efforts to dissect the regulatory pathways involved in latency, transformation, and lytic switch.

## 2. Materials and Methods

### 2.1. Cell Culture

CEF used in this study were prepared from 10-day old Valo SPF embryos. Cells were cultured in M199 medium (Thermo Fisher Scientific, Paisley, Scotland, UK) supplemented with 5% fetal bovine serum (FBS, Sigma, St. Louis, MO, USA), 100 units/mL of penicillin and streptomycin (Thermo Fisher Scientific), 0.25 µg/mL Fungizone (Sigma), 7.5% sodium bicarbonate, and 10% tryptose phosphate broth (Sigma). The MDV-transformed LCLs MSB-1 [40] from a spleen lymphoma induced by the BC-1 strain of MDV and HP8 [41] from a GA strain-induced tumor were grown at 38.5 °C in 5% CO_2_ in RPMI 1640 medium (Thermo Fisher Scientific) containing 10% fetal bovine serum, 10% tryptose phosphate broth, 1% sodium pyruvate solution (Sigma), and 100 units/mL of penicillin and streptomycin.

### 2.2. gRNAs and GFP Donor Template

A dual gRNA construct pp38-gNC, which expresses two gRNAs targeting both ends of *pp38* gene and Cas9 nuclease in pX330A-1 × 2 vector was used for *pp38* deletion in MSB-1 [39]. Two-part guide RNA system containing crRNA:tracrRNA guide complex was used for HP8 editing. The same gRNA sequences for pp38-gN and pp38-gC were used for synthetic crRNAs production by Integrated DNA Technologies (IDT, Diego, CA, USA). The 36-mer crRNA contains a variable gene-specific 20-nt target sequence followed by 16-nt sequence that base-pairs with the tracrRNA. The 67-mer tracrRNA contains the gRNA-scaffold sequence as well as the 16-nt sequence complementary to crRNA. The lyophilized crRNA and tracrRNA pellets were resuspended in Duplex buffer (IDT) at 200 μM concentration and stored in small aliquots at −80 °C. GFP expression cassette used for insertion into *pp38* was released by PacI restriction digestion from pGEM-sgA-GFP [42] and purified by gel extraction kit (Sigma) after separation with agarose gel.

### 2.3. Generation of Stable MSB-1 and HP8 Cell Lines Expressing Cas9

We used NEPA21 Electroporator for transfection of MDV-transformed LCLs. 1 × 10^6^ of MSB-1 or HP8 cells were resuspended in 96 μL Opti-MEM medium (Thermo Fisher Scientific) and mixed with 10 μg of pX459-V2.0 in 4 μL Opti-MEM to make a total volume of 100 μL and electroporated with optimized condition at voltage 175 V and a pulse width 1 ms of poring pulse for MSB-1 and at voltage 275 V and a pulse width of 1.5 ms of poring pulse for HP8. At 48h post electroporation, the transfected cells were selected with puromycin (Sigma) at a concentration of 1 µg/mL. After selection, single cell clones were isolated by sorting and Cas9 expression was assessed by western blotting with anti-Flag antibody.

### 2.4. Generation and Characterization of pp38 Deletion Cell Line HP8-Δpp38

1 × 10^6^ of HP8-Cas9 cells were resuspended in 96 μL Opti-MEM medium. Two crRNAs pp38-gN and pp38-gC were mixed with equal molar of tracrRNA to a final duplex concentration of 100 µM in 4 µL of duplex buffer and incubated at 95 °C for 5 min. After the crRNA and tracRNA duplex was allowed to cool to room temperature, it was mixed with cell suspension and electroporated with the conditions for HP8 cells. At 24 h post electroporation, 1 × 10^5^ cells were harvested and analyzed by PCR. At 48 h post electroporation, single cells were sorted into 96 wells. After 7 days incubation, cells were collected and analyzed by PCR. The harvested cells for PCR analysis were lysed in 1× Proteinase K based DNA extraction buffer (10 mM Tris-HCl, pH 8, 1 mM EDTA, 25 mM NaCl, and 200 µg/mL Proteinase K) at 65 °C for 30 min. Extracted DNA template of 1 µL was used for PCR with primers outside the targeted sites to identify the correct *pp38* gene knocked-out [39].

### 2.5. Reactivation of MDV from MSB-1-Δpp38

1 × 10^6^ of MSB-1 or mutant clones C39 and D4 were co-cultivated with CEF monolayers for 24 h and then removed. CEF monolayers were fixed with 4% paraformaldehyde and permeabilized with 0.1% Triton X-100 after 5 days incubation. The expression of pp38 was evaluated by immunofluorescence assays (IFA) using fluorescence microscopy. The cells were stained with monoclonal antibody (Mab) BD1 (generated at Pirbright Institute, Woking, UK) for pp38 expression and HB3 (generated at Pirbright Institute) for gB expression as a control. Images were taken using a Leica DM IRB microscope (Leica Microsystems, Wetzlar, Hesse, Germany).

### 2.6. Western Blotting Analysis

Expression of pp38 in MSB-1-Δpp38 and HP8-Cas9-Δpp38 cells before and after sodium butyrate (NaB) or 5-Azacytidine (AZA) treatment was determined by western blot analysis using anti-pp38 Mab BD1 as the primary antibody. Mab against MDV Meq (FD7, generated at Pirbright Institute) and α-tubulin (Sigma Aldrich) were used as a control. Briefly, 1 × 10^6^ cells were collected before and after treatment with 0.5 mM NaB (Sigma) or 10 µM AZA for 72 h and boiled with TruPAGE^TM^ LDS sample buffer (Sigma) for 10 min. The samples were separated on a 4–12% TruPAGE^TM^ Precast Gel, and the resolved proteins were transferred onto polyvinylidene difluoride (PVDF) membranes. Immunoblots were blocked with 5% skimmed milk, and then incubated with anti-pp38 and anti-Meq antibodies. After probing with primary antibodies, the blots were incubated with secondary antibody IRDye^®^ 680RD goat anti-mouse IgG (LI-COR, Lincoln, NE, USA) and visualized using Odyssey Clx (LI-COR). For α-tubulin detection, the PVDF membranes were incubated in stripping buffer (100 mM 2-Mercaptoethanol, 2% SDS, 62.5 mM Tris-HCl PH 6.7) for 30 min at 50 °C, and then blocked with 5% skimmed milk after washing in PBS twice for 10 min. The same procedure was followed for detection of α-tubulin and visualized by Odyssey Clx (LI-COR).

### 2.7. Growth of HP8-Cas9-Δpp38 Cells

The growth of HP8-Cas9-Δpp38 cells along with non-edited HP8-Cas9 cells were monitored by IncuCyte S3 live cell imaging (Essen Bioscience Ltd., Hertfordshire, UK). Briefly, 8000 cells were seeded in a 96 well plate and images were captured every 2 h for 216 h from four separate regions per well using a 10× objective. By recording the phase object confluence, the growth of HP8-Cas9-Δpp38 clones were compared with parental HP8-Cas9. IncuCyte data were analyzed by two-way ANOVA (analysis of variance) with Tukey’s multiple comparisons using GraphPad Prism version 7.01 (GraphPad Software, Inc., San Diego, CA, USA). The results were shown as mean ± standard error (SE) from four replicates each with 4 separate regions per well representative of three independent experiments.

### 2.8. Single Cell Sorting and Flow Cytometry Analysis

For single cell cloning, cells were washed twice with PBS containing 1% FBS and centrifuged at 450× *g* for 5 min at room temperature. The cell pellets were suspended in cold PBS/5%FBS and sorted into 96 well plate U bottom with growth medium by fluorescence-activated cell sorting (FACS) using FACSAria II (BD bioscience, Wokingham, Berkshire, UK). After 2 weeks incubation, fluorescence intensity was then measured by flow cytometry analysis. Briefly, HP8-GFP cells were washed twice with PBS with centrifugation at 450× *g* for 5 min at room temperature, and then fixed with 4% paraformaldehyde before washing with PBS. GFP expression was determined by using a flow cytometer, MACSQuant^®^ analyser (Miltenyi Biotec, Woking, Surrey, UK).

### 2.9. q-PCR for GFP Copy Number

DNA was extracted from 2 × 10^6^ cells using the DNeasy 96 Blood and Tissue kit (Qiagen, Hilden, North Rhine-Westphalia, Germany) for real-time qPCR to determine GFP copy number. Duplex real-time qPCR carried out to detect the GFP gene and chicken ovotransferrin gene enabled calculation of GFP copies per 10,000 cells using a dilution series of pCDNA3-EGFP DNA and p-GEM-T-ovo [43] to produce standard curves. The details of the primers, which include MF/MR for GFP and ovoF/ovoR for ovotransferrin gene are listed in Table 1. PCR amplification was carried out in a 20 μL reaction volume with 10 μL of PowerUp SYBR Green Master Mix (Thermo Fisher Scientific), 0.5 μM forward and reverse primers, and 4 μL extracted DNA. The PCR conditions used were: 95 °C for 2 min, followed by 40 cycles at 95 °C for 15 s and 60 °C for 1 min. All qPCR tests were run in triplicate on the ABI 7500 Fast Real-time PCR System (Thermo Fisher Scientific).

## 3. Results

### 3.1. Editing of pp38 in MSB-1 Cells

**Figure 1:**
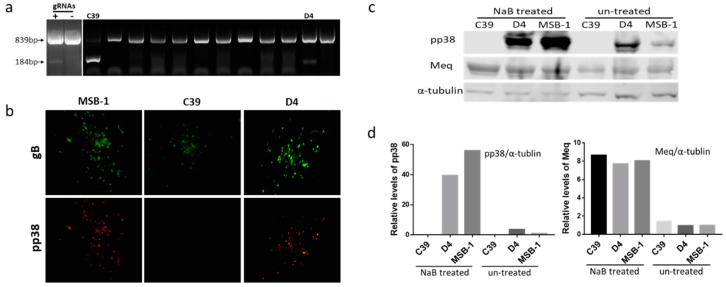
Deletion of *pp38* gene by CRISPR/Cas9 editing in MSB-1 cell. (**a**) PCR amplification of the edited region, using primers NF and CR on the cell lysates of transfected cells at 24 h post electroporation and single cell clones after sorting. The top band with un-edited/partially-edited sequence with small indels was expected to be around 839 bp, while deletion of the sequence between the Cas9 cleavage sites would result in a 184 bp PCR product. The two clones (C39 and D4) with 184 bp band were indicated. (**b**) Confirmation of the *pp38* gene deletion in MSB-1 by IFA on plaques formed by co-cultivation of edited MSB-1 clones on chick embryo fibroblasts (CEF) monolayer with anti-pp38 monoclonal antibody BD1 (red), anti-gB monoclonal antibody HB3 (green) staining was used as an infection control. Pictures were taken with 100× magnification. The data shown are representative of three independent experiments. (**c**) Detection of pp38 expression by western blotting with anti-pp38 monoclonal antibody BD1 and anti-Meq monoclonal antibody FD7 before and after NaB treatment on MSB-1 and edited clones C39 and D4. For the loading control, the same blot was stripped and re-probed with anti-α-tubulin antibody. The data shown are representative of three independent experiments. (**d**) Relative signal intensities of the pp38 and Meq western blot band were quantified using ImageQuant and normalized against the corresponding signal from the α-tubulin band. The signal from the untreated control MSB-1 cells was set as 1.

Previously, we have demonstrated that CRISPR/Cas9 editing can be applied efficiently in targeted editing of the MDV genome during lytic stages of infection in infected CEF cultures [37,39]. Based on this success, we set out to explore whether we could carry out targeted editing of the largely latent MDV genome in the transformed cells. We chose to target the pp38 gene in the MDV-transformed cell line MSB-1 using the same gRNAs that were successfully used for pp38 deletion from CVI988 genome in CEF culture system [39]. In the hard-to-transfect T cell line MSB-1 cells, with a predominantly latent genome, we wanted to assess the editing efficiency. To delete pp38 in MSB-1 cells, we first transiently transfected dual gRNA construct pp38-gNC, which expressed Cas9 nuclease and the two gRNAs that targeted both ends of the pp38 gene into MSB-1 cells. PCR tests on the genomic DNA from cells harvested 24 h after transfection using pp38-specific primers located at the flanking region of Cas9 targeting sites [39] clearly demonstrated the expected two bands, suggesting successful editing of the locus. The top band with 839bp sized PCR product represented unedited or edited target site/s with small indels. The bottom band with 184bp product corresponded to the edited sequence, which represents the flanking sequence of the deleted region between the two Cas9 cleavage sites in pp38 (Figure 1a). The top band being predominant indicated either the relative low editing efficiency or that the two sites were not cleaved simultaneously. Indeed, most MD lymphoma-derived cell lines have multiple copies of the MDV genome integrated in different chromosomes of chicken genome [1,3,7]. Simultaneous editing of multiple targets is a challenge and may have contributed to the lower editing efficiency of the pp38 locus observed in this experiment. Moreover, the lower transfection efficiency of LCL compared to the CEF may also have affected the efficiency. This was investigated by single cell sorting to analyze the editing pattern at a single cell level. As shown in Figure 1a, most clones only showed the top unedited/partially edited band, most likely due to the low transfection efficiency of the LCLs. The appearance of both bands from two clones indicated editing of the locus, although not all of the copies in the same cell may have been targeted. Differences in the predominance of the bottom (clone C39) or the top (clone D4) bands between the two clones probably suggest the differences in the editing efficiencies. Indeed, sequence analysis of the bottom band confirmed the deletion between the two Cas9 cleavage sites in pp38. We also cloned the ~839-bp top band of both clones into pGEM-T-vector, and sequence analysis confirmed the presence of the mixed population of wild type and mutated sequences in both clones. Of the 20 individual sequences examined from each of the clones, C39 clone showed 19 edited sequences compared to the 15 in the clone D4. All the mutated *pp38* sequences analyzed were targeted by either one or both gRNAs that resulted in indel mutations causing pre-mature stop codon or frame shift [44] Although the wild type *pp38* was still present at a small percentage, the sequencing and pp38 staining results (Figure 1b) did confirm the successful editing of *pp38* in the MSB-1 cell line.

These two clones were assessed further in a virus reactivation assay by co-cultivation of 10^6^ cells with CEF monolayers to examine the development of MDV plaques from reactivated viruses. Around 10 plaques were observed in each well after co-cultivation. Plaques formed on CEF from both C39 and D4 clones expressed glycoprotein B as detected by the anti-gB monoclonal antibody HB3 (Figure 1b). However, pp38 expression (detected with anti-pp38 antibody BD1) was seen only on the plaques arising from the co-cultured D4 clone. We reasoned that this is likely to be related to the near complete editing of the *pp38* loci in the C39 clone, compared to the D4, where the unedited *pp38* sequences that may still be present express pp38. All the MDV plaques produced on CEF following the co-culture of clone C39 were pp38 negative in spite of the detection of top 839-bp upper band representing the wild type locus by PCR analysis.

MDV-encoded pp38 is considered to be one of the best biomarkers of the latency-to-lytic switch in the LCL, where most of the MDV genome is held in a latent state, thought to be through epigenetic mechanisms [30]. However, previous studies have shown that between 1–10% of the MSB-1 population expressed pp38 at low levels [31,40]. Lytic replication in LCL can be induced with histone deacetylase (HDAC) inhibitors such as NaB or methylation inhibitor AZA [30]. In order to examine further whether the edited clones did express pp38, we treated the cells with NaB at 2.5 mM concentration, before assessing the pp38 expression by western blotting analysis using pp38-specific antibody BD1. These experiments showed that the pp38 expression increased dramatically after NaB treatment in both the wild type MSB-1 and partially edited clone D4 (Figure 1c,d). In contrast, no pp38 expression was evident before and after the NaB treatment in the C39 clones, further confirming that it represented a total deletion of the *pp38* loci in the MSB-1 cells. Interestingly, Meq was consistently expressed in wild type MSB-1 and the two clones, although the levels did increase following the induction of lytic replication after NaB treatment.

### 3.2. Editing of pp38 in HP8 Cell Line

**Figure 2:**
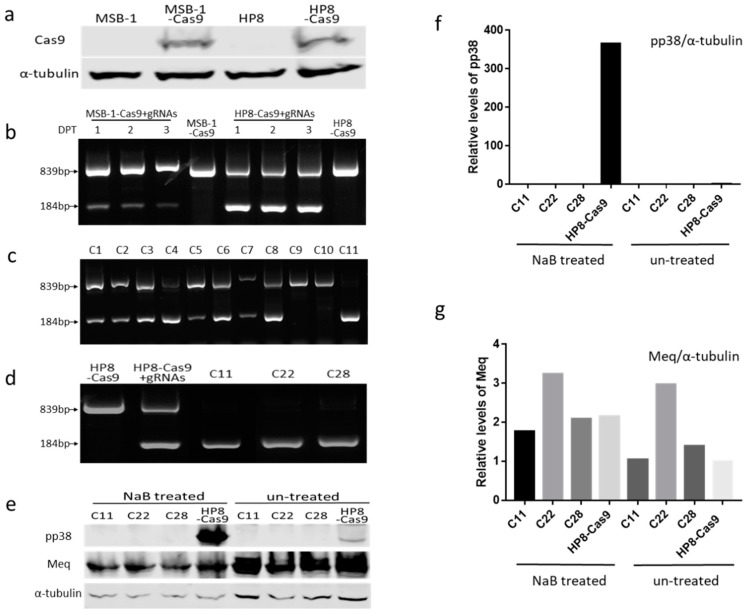
Deletion of the *pp38* gene by CRISPR/Cas9 editing in HP8 cells. (**a**) Detection of Cas9 expression on single clones of MSB-1 and HP8 cell lines stably expressing Cas9 by western blotting. Cell lysates of MSB-1-Cas9 and HP8-Cas9 along with MSB-1 and HP8 were separated by SDS-PAGE, Western blotted, and probed with anti-Flag antibody, α-tubulin was used as the loading control. (**b**) PCR amplification of the edited region, using primers NF and CR on the cell lysates of transfected cells at 1, 2, and 3 days post transfection. DPT, days post transfection (**c**) PCR amplification of the *pp38* locus on isolated single cell clones of transfected HP8-Cas9 with two part gRNA system showing the two bands. (**d**) PCR results of selected clones used for subsequent analysis. The unedited HP8-Cas9 and the mixed population after editing (HP8-Cas9 + gRNAs) were also included. (**e**) Detection of pp38 expression by western blotting with anti-pp38 monoclonal antibody BD1 and anti-Meq monoclonal antibody FD7 before and after NaB treatment on HP8-Cas9 and the edited clones. For the loading control, the same blot was stripped and re-probed with anti-α-tubulin antibody. (**f**) and (**g**) Relative signal intensities of the pp38 (**f**) and Meq (**g**) western blot band were quantified using ImageQuant and normalized against the corresponding signal from the α-tubulin band. The signal from untreated control HP8-Cas9 cells was set as 1.

Having demonstrated that the *pp38* sequence can be edited in MSB-1 cells by transient transfection of plasmid DNA, albeit at a relatively low efficiency, we explored the efficacy of using synthetic gRNAs with a two-part guide RNA system (IDT) into stable Cas9 expressing MDV cell lines. For the generation of stable Cas9 expressing cell lines, MSB-1 and HP8 cells were transfected with pX459-V2 and selected with puromycin. Single cell clones isolated by FACS were examined for Cas9 expression by western blot analysis with anti-Flag antibody. Figure 2a shows Cas9 expression in the selected MSB-1 and HP8 clones, which were used subsequently for MDV genome editing. Approximately 1 × 10^5^ MSB-1-Cas9 and HP8-Cas9 cells transfected with 100 µM of each gRNA were harvested every day for three days post-transfection to assess the editing efficiency by PCR using the same pair of primers located at the flanking region of Cas9 targeting sites. Two bands representing the wild-type/partially-edited and the edited gene (839 bp and184 bp, respectively) could be observed in both the cell lines (Figure 2b) demonstrating successful editing of the *pp38* loci in these populations. However, there were clear differences in the predominance of the wild-type/partially-edited or the edited bands between the two cell lines. While the wild-type/partially-edited top band was the predominant one in the MSB-1 cells, HP8 cells mostly demonstrated the edited bottom band, suggesting more efficient editing in the HP8 cells. Moreover, the strength of the edited bottom band in MSB-1 appeared to decrease over time suggesting the gradual elimination of the edited population. This was also evident from the unhealthy morphology of the MSB-1 cells when viewed under the microscope after being edited [45]. In contrast, the transfected HP8 cells continued to show robust edited bottom band over the three day period and appeared to maintain a normal healthy morphology.

Single cell clones of transfected/edited HP8 cells were sorted and analyzed by PCR to assess the editing pattern. Out of the 144 single cell clones analyzed, 51 showed only the wild-type/partially-edited top band, 83 showed both bands representing incomplete editing, while 10 showed no detectable top band suggesting the complete editing of *pp38*. Figure 2c shows the examples which covered all three editing patterns, C1–C8 representing mixed partially edited clones, C9–C10 showing the un-edited/partially-edited clones and C11 being completely edited. Three fully-edited clones (C11, C22 and C28) selected for further studies (Figure 2d) confirmed the potential for efficient editing of the *pp38* loci in HP8 stably expressing Cas9. For further confirmation of the knock-out of pp38 from the selected HP8 clones, pp38 expression was assessed before and after treatment with either NaB or AZA. As expected, pp38 level increased dramatically after treatment in HP8-Cas9, whereas no pp38 expression could be observed in the deleted clones before and after NaB (Figure 2e) or AZA (Supplementary Figure S1) treatment. In contrast, Meq was expressed in all the clones before and after drug treatment although the expression level showed variations between samples. This further confirmed the successful *pp38* knockout in HP8 cells (Figure 2e–g and Supplementary Figure S1).

**Figure 3:**
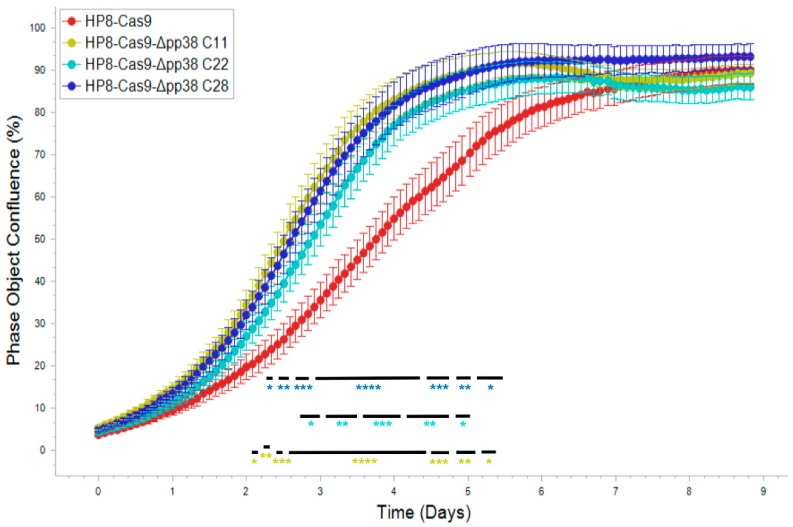
Proliferation of the HP8-Cas9 and the *pp38*-deleted clones monitored in real time using IncuCyte S3 live imaging system. Cell phase object confluence of each cell population was determined every 2h for 216 h from four separate regions per well and four wells per sample by IncuCyte S3 and compared with HP8-Cas9 control. Growth curves are shown as mean ± standard error (SE) representative of three independent experiments. Asterisk (*) indicates statistically significant differences between *pp38* deleted clones and parental HP8-Cas8 cells at different times. *, *p* < 0.05; **, *p* < 0.01; ***, *p* < 0.001; ****, *p* < 0.0001.

In a previous study using antisense oligonucleotides transfected into LCL, pp38 was suggested to be important for the maintenance of the transformed phenotype [46]. To further explore the role of pp38 in LCL with a much accurate gene editing approach, we examined the effect of deletion of *pp38* on the proliferation of HP8 cells. For this, we carried out kinetic monitoring of proliferation of the wild type HP8-Cas9 and the *pp38* deleted clones C11, C22, and C28 using IncuCyte S3 Live-Cell Imaging system. The cell proliferation data in real time from the images collected at 2 h intervals showed that all the *pp38*-deleted clones proliferated at a significantly higher rate between 2–6 days compared to parental HP8-Cas9 cells. The increased growth lasted for two (C22) to three (C11 and C28) days before reaching the plateau phase (maximal usage of surface space) (Figure 3). These results suggested that expression of pp38 was not essential for the continued proliferation of these transformed cells.

### 3.3. Insertion of Marker Gene into MDV Genome in HP8 Cells by NHEJ Pathway

**Figure 4:**
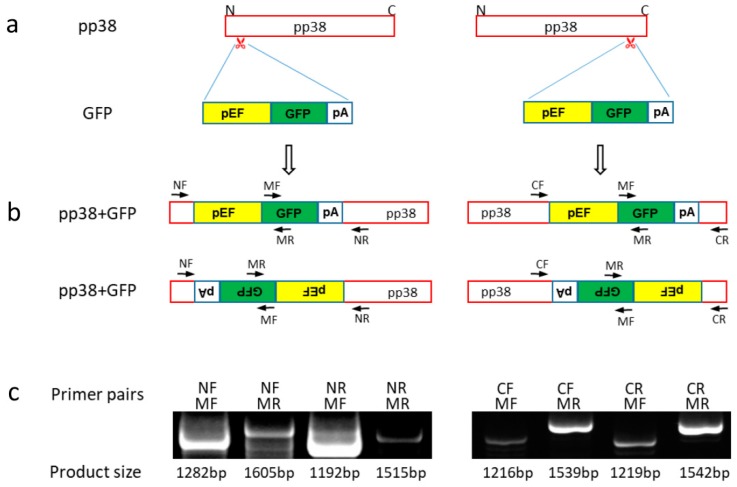
Knock-in of green fluorescent protein (GFP) expression cassette into *pp38* of HP8-Cas9. (**a**) Schematics of GFP expression cassette and *pp38* with the gRNA targeting sites at N and C terminus. (**b**) Schematics of the anticipated *pp38* with inserted GFP expression cassette. GFP could be inserted in either orientation. As a result, there are two potential products for each target site. The location of primers used to confirm the presence and the orientation of GFP insert by junction PCR are shown. (**c**) Junction PCR products with the expected size of the mixed GFP positive cells using the primers shown in Figure 4b. GFP was successfully inserted into *pp38* at both sites with both orientations.

Since *pp38* can be deleted from the MDV genome in LCL without affecting their replication, we wanted to examine whether gene editing approach could be used to insert a fluorescent gene into the *pp38* locus as a biomarker, particularly for studies involving latency to lytic switch of MDV. For the proof of concept, we inserted GFP into the *pp38* locus by co-transfection of GFP expression cassette along with the gRNA targeting either 5′ or 3′ end of *pp38* in the HP8-Cas9 cell line. GFP expression cassette is in the format of the DNA fragment released from the plasmid pGEM-sgA-GFP by PacI restriction digestion (Figure 4a). Two days post-transfection, we observed GFP-positive cells from both transfections. These GFP-positive cells were then sorted and the evidence of successful insertion of GFP at the desired location was analyzed by junction PCR with primers located at either end of the flanking sequence of target sites together with another primer from GFP. The primer sequences used for junction PCR are listed in Table 1. As GFP could be inserted in either orientations, both possibilities were checked with corresponding primers as indicated in Figure 4b. PCR products of the expected size for both orientations at both target sites were detectable (Figure 4c), indicating that GFP expression cassette was successfully inserted in the target loci in both orientations.

**Figure 5:**
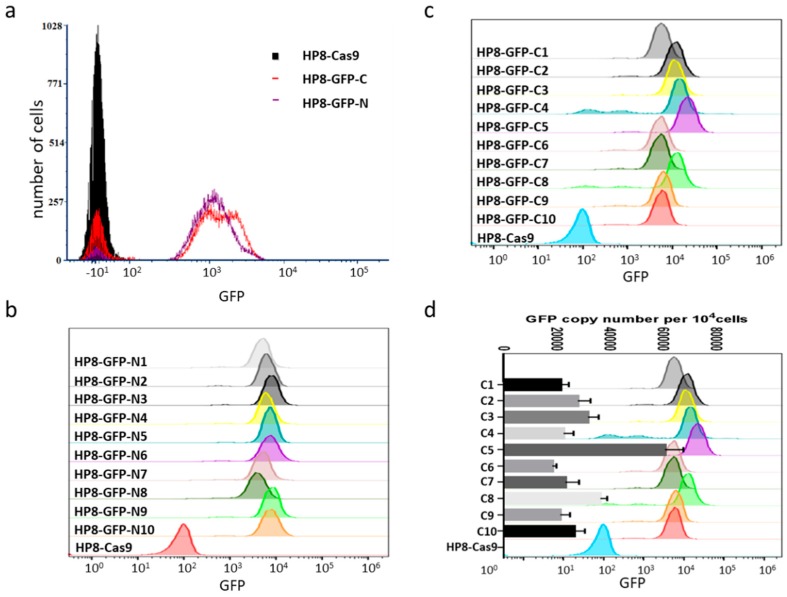
GFP expression in HP8-Cas9 cell line. (**a**) fluorescence-activated cell sorting (FACS) analysis of GFP expression on HP8 cells with GFP inserted in *pp38* locus at N (HP8-GFP-N) and C (HP8-GFP-C) terminus. (**b**) FACS analysis of GFP expression on expanded population of 10 single cell clones of HP8-GFP-N after two weeks incubation. Fluorescence intensity varies between different clones. (**c**) FACS analysis of GFP expression on expanded population of 10 single cell clones of HP8-GFP-C after two weeks incubation. Fluorescence intensity varies between different clones. (**d**) GFP copy number per 10^4^ cells was measured on 10 single cell clones of HP8-GFP-C by q-PCR and plotted against the GFP fluorescence intensity measured by FACS analysis. The chicken ovotransferrin gene was used for calculation of GFP copies per 10^4^ cells.

During cell sorting for GFP positive cells by flow cytometry, it was observed that the intensity of fluorescence varied between cell populations and between cells within the same population (Figure 5a). We compared the fluorescence intensity of 10 isolated clones for each insertion site. Indeed, there was variation in fluorescence intensity between different clones for both populations as shown in Figure 5b,c. As there were multiple copies of *pp38* present in one cell, we reasoned that the different fluorescence intensity is likely to be a reflection of the different number of GFP copies inserted into *pp38*. To investigate this hypothesis, we determined GFP copy numbers per 10,000 cells by qPCR on 10 HP8-GFP clones with GFP inserted at the C-terminus, which showed more variations on fluorescence intensity. GFP copy number determined by qPCR was indeed in agreement with flow cytometry data, which showed varied intensity of fluorescence with clone 5 having the highest GFP copy number and highest fluorescence intensity (Figure 5c,d). These results demonstrated that GFP expression cassette can be inserted into the *pp38* locus and the GFP expression levels correlated with the copy numbers.

## 4. Discussion

A unifying and important principle for all herpesviruses is to establish latency for prolonged, usually life-long, maintenance of the genetic material of this group of pathogens in infected hosts. During latency, only very few viral genes express with the sole purpose of genome maintenance and the avoidance of a fully lytic replicative cycle resulting in cellular death [47]. Latency is a poorly understood state with the involvement of a complex set of regulatory factors and events. Studying the latency in MDV-transformed LCL has been a challenge mainly because of the lack of tools for in situ manipulation of the viral or host genes. In the last few years, CRISPR/Cas9-based gene editing has been used to investigate the factors involved in latency in a number of herpesvirus infections [35,48]. Following the successful application of CRISPR/Cas9 system on herpesvirus of turkeys (HVT) genome editing for gene disruption [36], knock-in of foreign genes for recombinant vaccine development [37], and targeted gene knock-out in MDV genome for gene function studies [39]. We report here the first use of this technology to introduce targeted mutations in situ into the viral genome of MDV-transformed LCLs. Using the same gRNAs used for *pp38* deletion in infected CEF, we show that non-homologous end joining (NHEJ) repair pathway can also be used to generate defined gene knockout and to insert the specific sequence into the viral genome in MDV transformed cell line. To our knowledge, this is the first study to demonstrate effective use of the CRISPR/Cas9 system for viral genome manipulation in MDV-transformed cell line for virus-host interaction and latency studies.

For the *pp38* deletion in MSB-1 cells, the efficiency with transient plasmid transfection method was very low possibly due to much lower transfection efficiency despite using the NEPA21 electroporator with optimized conditions [39]. This was evident from the low recovery rate of *pp38*-edited clones. Only two out of two hundred cell clones screened contained the small edited band. It is also possible that most of the edited cells were dying off and there was less chance to recover the edited population by single cell cloning, as was evident from the observation of cell death in the edited mixed population as detected by PCR with two-part gRNA system. The bottom band, representing the cell population with *pp38* deletion was gradually becoming weaker with continued passage post transfection (Figure 2b). Furthermore, the two edited clones showed different editing efficiency, which is reflected by the predominance of the bottom band in C39 and top band in D4, respectively. MDV is known to integrate its genome in the telomeric region of multiple chromosomes [1,3,7] with some of the RB-1B induced tumor cell lines showing integrations in 5–6 chromosomes as determined by FISH [49]. Considering the presence of multiple copies of MDV genome in the LCL, it was not surprising that not 100% of the *pp38* sequences in the same cell were edited. In contrast, the editing efficiency was much higher when the synthetic gRNAs were delivered into cells stably expressing Cas9. This reflects that the delivery of the small sized gRNAs is more efficient compared to the large sized Cas9/gRNA expression plasmid.

MDV genome consists of the unique long (U_L_) and short (U_S_) regions flanked by terminal and internal repeat long (TR_L_/IR_L_) and short (TR_S_/IR_S_) regions. The *pp38* gene spans the junction between the internal repeat and the unique long region [13,50]. Although the method described here for editing of *pp38* in the MDV genome was highly successful, there is the need for increasing the efficiency, especially for high throughput approaches. This is also important for the editing of the genes in the repeat regions where the copy number of the corresponding genes is doubled. Cas9 and gRNAs delivery by lentiviral vectors has been considered being highly efficient for gene transfer. However, whether it is a more efficient way for MDV genome editing in MDV transformed LCL in comparison to the described approach here remains to be tested. The key point for successful deletion of defined sequence is that two gRNAs have to cleave the target sequence simultaneously. The sequence of the top band showed that some of them displayed mutations at the gRNA target site, rendering them resistant to subsequent CRISPR/Cas9 editing. Although more gRNAs could be used to increase the efficiency of editing, it is important to confirm the deletion of the target gene by examining protein expression using assays such as western blotting and IFA. For example, DNA from the clone C39 showed low levels of unedited band by PCR, yet failed to recover any pp38-positive plaques. Although the reasons for this is not clear, it is possible that not every single copy of the MDV genome sequence from LCL can be reactivated. In terms of differences in the ease of transfection and recovery rate after editing between MSB-1 and HP8 LCLs, it could be the result of presence of both MDV-1 and MDV-2 viruses in MSB-1 cells, whereas only MDV-1 is present in HP8 cells. Hence, HP8 is deemed a better choice not only for the cleaner background also for higher transfection efficiency and recovery rate.

Phosphoprotein pp38 is a unique MDV gene shown to be involved in early cytolytic infection in B lymphocytes but not in the feather follicle epithelial cells [38]. It has been shown to be not essential for the induction of tumors using deletion mutant viruses [20]. Xie et al. have suggested the involvement of pp38 in maintenance of transformation of LCL by using oligonucleotides complementary to the translation initiation site of *pp38* [46], although this claim is questionable as no data on the expression levels of pp38 following oligonucleotide treatment was available. In contrast, the results obtained here clearly show complete absence of pp38 expression in the deleted cell lines, even after induction with NaB and AZA. Interestingly, cell proliferation rates of the *pp38*-deleted clones showed an increase for 2 to 3 days at the log phase, although no differences were observed after the cell densities reached the plateau. Similar results were also obtained for *pp38* deleted MSB-1 clone C39 [45]. These results clearly demonstrated that pp38 is not essential in maintaining the transformed phenotype as shown by the continued proliferation. Although the off-target effect is a concern for CRISPR/Cas9 editing, the increased proliferation of edited clones is unlikely due to the off-target effect as sequence analysis of previously edited HVT viral genome by PacBio sequencing showed no off-target effect [51]. A previous study has also suggested a role for pp38 in maintaining transformed phenotype in vivo by blocking apoptosis [38]. While the increased proliferation of the *pp38*-deleted LCL may appear to be contradictory to this finding, our studies were carried out in situ in established LCL, which may have acquired other mutations related to the apoptosis pathways. MDV-encoded proteins pp38 and pp24 share the same genome sequences at the amino-terminal ends and hence pp38-gN gRNA will also be able to target pp24 gene for editing. Disruption of the pp24 locus in these cells was verified by sequencing, and the lack of protein expression confirmed by western blot analysis [44,45]. Because of deletion of both *pp38* and pp24, it is difficult to attribute the phenotype of increased proliferation of mutated HP8 cells to any one of these two proteins. Nevertheless, our studies clearly demonstrated that neither of these proteins are essential for the continued proliferation of the MDV-transformed HP8 cells. Although a generation of mutant viruses is valuable to study gene function in vitro and in vivo using virus infection models, the role of individual MDV genes in maintaining the transformed phenotype of tumor cells can best be studied in MDV-transformed LCL as described here. Thus, our studies demonstrate the value of CRISPR/Cas9-based editing as a powerful tool to introduce the targeted mutations in situ into the MDV-transformed LCLs.

The NHEJ repair pathway has been used here to generate defined gene knockout by eliminating the sequence between two Cas9 cleavage sites. The NHEJ pathway also allows insertion of specific sequences such as GFP at the defined locations, enabling the generation of marker viruses. As a proof-of-principle, we have shown the insertion of GFP into the *pp38* locus in MDV-transformed LCLs with two target sites. As pp38-gN also targets pp24, the GFP expression cassette could be inserted into either *pp38* or pp24 when pp38-gN was used. In either case, the PCR result would be the same. Interestingly, this experiment also demonstrated the potential effects of the differences in the copy numbers on the expression levels of the reporter marker GFP. Further modifications to this approach can be developed to fit the different scenarios and purposes for the targeted engineering of MDV genome in situ in the LCL. For example, by including the homology arms in the donor template, the described workflow can utilize homology directed repair (HDR) to knock-in specific sequences and allow more precise editing such as epitope tagging or fluorescent protein fused to a viral protein, albeit at lower frequencies. MDV-1 has a two-phase life cycle, consisting of a lytic and a latent phase, the latter closely associated with the oncogenesis of the virus, yet the underlying molecular mechanisms of cell transformation remain unclear. The major questions such as the factors that maintain the latency of the virus and how the virus is reactivated from the latent state are to be answered. CRISPR/Cas9-mediated editing of the MDV genome in MDV-transformed LCLs described here will provide a new platform for study of MDV-induced oncogenesis. In particular, it will facilitate rapid analysis of the roles of individual MDV genes and host genes in latency, transformation, reactivation, and host-virus interactions.

## Figures and Tables

**Table 1:** List of primer sequences.

Primer	Sequence (5′-3′)
NF	TTGGAATAGCCCCCTTCCCC
NR	TTCGAAGCAGAACACGAAGGG
CF	GATTCCACCTCCCCAGAATCC
CR	CAGAGAATGCAACAATGCGT
MF	ATGGTGAGCAAGGGCGA
MR	CCGGTGGTGCAGATGAAC
ovoF	CACTGCCACTGGGCTCTGT
ovoR	GCAATGGCAATAAACCTCCAA

## Supplementary Materials

The following are available online at https://www.mdpi.com/1999-4915/11/5/391/s1, Figure S1: Induction of *pp38* gene expression by AZA treatment in *pp38* deleted and un-edited HP8 cells.
