# Supplementary material for: Targeted Editing of the pp38 Gene in Marek’s Disease Virus-Transformed Cell Lines Using CRISPR/Cas9 System
Source: Viruses. 2019 Apr 26;11(5):391. doi: 10.3390/v11050391 (PMC6563304; doi:10.3390/v11050391)
Supplement: Supplementary file 1 [file viruses-11-00391-s001.pdf]

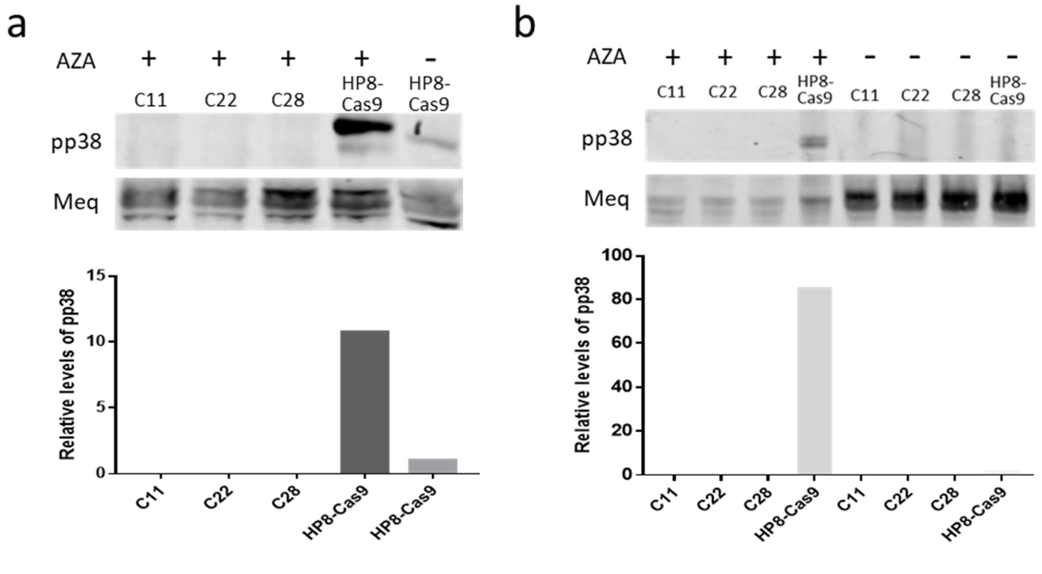

Supplementary Figure 1. Induction of pp38 gene expression by AZA treatment in pp38 deleted and un-edited HP8 cells. Detection of pp38 expression by western blotting with anti-pp38 monoclonal antibody BD1 and anti-Meq monoclonal antibody FD7 before and after AZA treatment at 10µM concentration on HP8-Cas9 and the edited clones. The experiment was carried out twice independently (a and b). The lower panel shows relative signal intensities of the pp38 western blot band quantified using ImageQuant and normalized against the corresponding signal from the Meq band. The signal from untreated control HP8-Cas9 cells was set as 1.
